# Advances in understanding COPD

**DOI:** 10.12688/f1000research.7018.1

**Published:** 2016-09-27

**Authors:** Gary P. Anderson

**Affiliations:** 1 Lung Health Research Centre, Faculty of Medicine, University of Melbourne, Parkville, VIC, Australia; 2 Department of Pharmacology and Therapeutics, Faculty of Medicine, University of Melbourne, Parkville, VIC, Australia

**Keywords:** Chronic Obstructive Pulmonary Disease, COPD, ACOS, COPD Asthma Overlap Syndrome, Bronchodilators, Myodebilitation, Catabasis

## Abstract

In recent years, thousands of publications on chronic obstructive pulmonary disease (COPD) and its related biology have entered the world literature, reflecting the increasing scientific and medical interest in this devastating condition. This article is a selective review of several important emerging themes that offer the hope of creating new classes of COPD medicines. Whereas basic science is parsing molecular pathways in COPD, its comorbidities, and asthma COPD overlap syndrome (ACOS) with unprecedented sophistication, clinical translation is disappointingly slow. The article therefore also considers solutions to current difficulties that are impeding progress in translating insights from basic science into clinically useful treatments.

## Introduction

Chronic obstructive pulmonary disease (COPD) is one of the most common serious lung diseases and is unenviably poised to remain the third most common cause of death worldwide well into the mid-century. Patients with COPD struggle with intractable breathlessness that results from airflow limitation, leading to pronounced disability. In practical terms, this means that patients, who characteristically ignore early warnings like reduced exercise capacity, become progressively less able to perform ordinary activities, even dressing and showering, without breathlessness (dyspnea). As the disease progresses, dyspnea may occur at rest and become intractable. Dyspnea leads to a profound loss in productivity and quality of life, alongside suffering, which is often compounded by recurrent chest infections and major comorbidities, especially affecting the heart and circulation, kidneys, metabolism, central nervous system (CNS), and the musculoskeletal system. Lung cancer is also common.

COPD is most commonly associated with exposure to inhaled irritants, especially cigarette smoking. However, the pattern of disease is changing markedly and as much as 30 to 40% of prevalent COPD may not relate to current smoking. In the recent CanCOLD (Canadian Cohort of Obstructive Lung Disease) study, a large Canadian population survey, 27% of all COPD was in never-smokers ^1^. Better educated and more affluent countries have seen dramatic falls in smoking prevalence in men and women. In poorer countries, adverse indoor air quality, massively increasing exposures to environmental inhaled pollutants, and an increasing number of children born with low lung function consequent to economic adversity are now emerging as major drivers of future COPD.

## New bronchodilators

In contrast to asthma—where airflow limitation is caused by excessive airway mucosal inflammation and bronchial smooth muscle contraction, both of which respond well to dual steroids-bronchodilator therapy—airflow limitation in COPD is characteristically poorly reversible. The reason for this difference is that COPD results from pronounced damage to the delicate architecture of the lungs that, at least with current technology, cannot be reversed, whereas in asthma the underlying lung architecture remains largely intact. Given that the structural basis of airflow limitation in COPD is currently intractable, pharmaceutical research has made the most progress in refining drug classes already known to be effective in COPD.

Both M3 cholinoceptor-favoring long acting anti-muscarinic (LAMA) and long-acting beta-2 adrenoceptor agonist (LABA) bronchodilators work alone or in combination in COPD to improve lung function, reduce gas trapping, and reduce exacerbations. The last several years have seen the introduction of a large number of new agents, and their combinations, for once- or twice-daily use, often and now controversially, co-formulated with an inhaled glucocorticosteroid (ICS) ^2– 6^. Broadly, LAMAs produce a slightly larger peak bronchodilator effect than LABAs, perhaps because inflamed COPD tissue produces *non-*neuronal acetlycholine in abundance ^7^ and the combination of adding a LABA to a LAMA adds about 30 to 40% more effect. New molecules combining both LABA and LAMA properties in a single molecule, so-called MABAs (dual muscarinic antagonist-beta 2 adrenoceptor agonists), are undergoing clinical trial but have not yet been approved. LAMAs have been shown to improve some aspect of tissue remodeling but there is clear evidence that this does not lead to an amelioration of lung function decline. Phosphodiesterase (PDE4) inhibitors, which like LABAs increase cAMP, improve forced expiratory volume in 1 second (FEV1), probably by reducing airways inflammation, but do not convincingly produce clinical bronchodilation.

Airway smooth muscle is neither hypertrophic or hyperplastic in COPD, unlike in asthma. Interestingly, taking the lead from severe asthma, a modified form of bronchial thermoplasty called “targeted lung denervation” (TLD), which uses a water-cooled probe tip to ablate nerve fibers rather than smooth muscle, has proven effective in several small and uncontrolled trials but it is not known whether this approach truly reduces cholinergic drive or interrupts afferent sensory fibers that contribute to the sensation of dyspnea ^8^.

## Chronic obstructive pulmonary disease molecular pathogenesis

It is also important to understand that “COPD” is an umbrella term that encompasses several distinct pathologies: emphysema, bronchiolitis, and bronchitis. The first of these, emphysema, is where the lung parenchymal tissue comprising the alveolar gas exchange units is progressively destroyed. This leads to reduced net surface area for gas exchange (measured clinically as the diffusing capacity to carbon monoxide, or DLCO), restricting oxygen uptake and carbon dioxide release. Emphysema also directly causes airflow limitation because microscopic elastin fibers in alveolar units operate as molecular “springs” by tethering the smallest airways open during expiration when compressive forces would otherwise tend to cause them to close. When these fibers are lost, the fragile small airways collapse, trapping gas in the lungs and in turn leading to overinflation, especially during exercise (dynamic hyperinflation) ^9^. Emphysema is itself heterogeneous and shows marked variation in clinical presentation, which is most likely genetically regulated ^10^.

**Figure 1.**
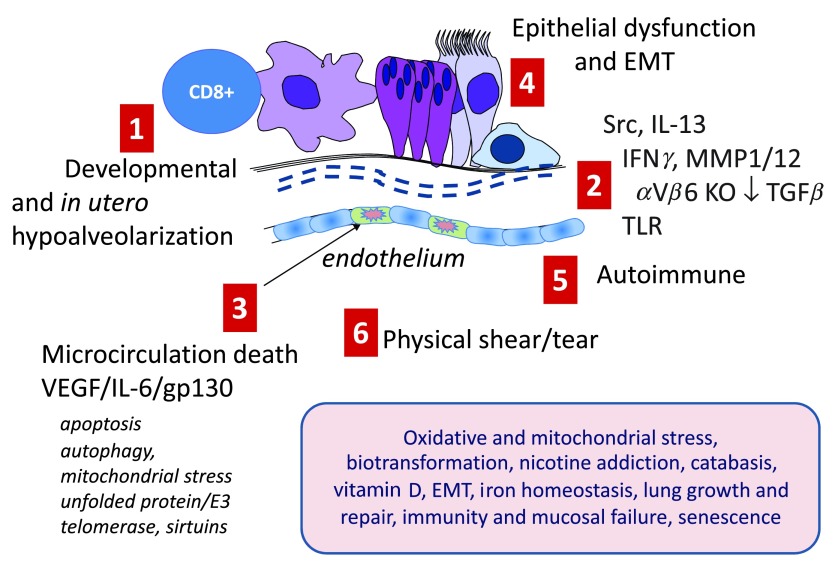
Major known mechanisms causing emphysema and small airway disease. The figure schematizes major pathways identified in experimental models which have reasonable human translational replication. ( **1**) *In utero* and early life insults that restrict lung growth and retard alveolization. ( **2**) A large group of mechanisms that integrate inflammation and defective immunity with destruction of elastin and collagen fibers required to maintain lung recoil and strength. These include dysregulation of SRC family kinases such as HCK, over-production of interferon gamma (IFNγ), and dysregulation of collagenases and elastases which are exemplified here as matrix metalloproteinase 12 (MMP12) and MMP1 but which also include neutrophil elastase and other serine proteases and defects in anti-proteases; serpinopathies, notably A1AT deficiency; dysregulation of the transforming growth factor beta (TGFβ)-mediated homeostasis; and defects in Toll-like receptor (TLR) receptor signaling. ( **3**) Defects in microcirculation leading to excessive apoptosis and alveolar endothelial cell death. For convenience, linked molecular processes, which are not restricted to the endothelium, are shown; these include dysregulation of AKT-mediated cell survival and apoptosis, cell and mitochondrial stress linked to autophagy, and protein damage and misfolding linked to E3-ligase-mediated destruction and accelerated molecular aging associated with defective sirtuin and telomerase activities. ( **4**) Defects in the epithelium (and progenitors including basal cells and stem niche cells) such as impaired CFTR function, ciliostasis, trans-differentiation toward a mucosecretory phenotype, and epithelial-mesenchymal transition (EMT). ( **5**) Autoimmune destruction of tissue. ( **6**) Structurally weakened lung tissue may literally rip when strained following hyperinflation and coughing. The shaded box denotes a wide group of known modifiers and cofactors found in genetic (genome-wide association study and eQT), profiling, and functional studies that predispose or amplify these driving processes: oxidative and mitochondrial stress leading to excessive oxidative damage; defective biotransformation (e.g., by cytochrome p450 isozymes and epoxide hydrolases of inhaled toxins in smoke and pollution that worsen injury); central nervous system genes controlling nicotine addiction, mainly the CHRNA3/5 nicotinic cholinoceptor; weakened catabasis/resolution; hypofunction of the vitamin D axis; EMT especially affecting small airways; iron homeostasis predisposing to infection and excessive oxidative stress; lung growth and repair; immunity and mucosal failure; and molecular aging and senescence. IL, interleukin; VEGF, vascular endothelial growth factor.

Murine models—despite the important caveats that mouse lung repairs emphysema well and has a less airway complex architecture than human lung—have identified multiple molecular candidate processes (see Figure 1). These include the following: (i) activation of nuclear factor kappa B (NFκB)-dependent and other forms of inflammation. Damage caused by inflammation is compounded by intense oxidant damage which is intensified by impaired protection from NRF2 (nuclear factor [erythroid-derived 2]-like 2, an anti-oxidant master switch) ^11, 12^ and depletion of anti-oxidants such as extracellular superoxide dismutase (SOD3) ^13^. These process may also trigger apoptosis often via the intrinsic stress response activation, which may involve the unfolded protein responses and excessive or unbalanced destruction of proteins by the E3-ligase:26S proteasome system ^14^; (ii) uncompensated cycles of inflammation and damage that destroy parenchyma in association with elastolysis (first observed in the 1970s ^15, 16^), intracellular autophagy and apoptosis driven by reduced endothelial survival factors and DNA damage, notably vascular endothelial growth factor ^17, 18^, and excessive ceramide ^19^. (iii) (dys)regulation of alveolar progenitor pool maintenance and reprogramming of macrophages toward pathogenic rather than pro-resolution phenotypes ^20, 21^; (iv) accelerated senescence (aging) associated with activation of p21 and telomere shortening ^22^; (v) autoimmune and auto innate processes concomitant with the emergence of bronchus-associated lymphoid tissue (BALT) and auto-antibodies that may be linked with aberrant interleukin-23/17 (IL-23/17) over-production, which is possibly linked to inflammasome activation itself ^23, 24^; (vi) acquisition of mutations in the stem cell/progenitor pool that alter daughter cell phenotype and function ^25^; (vii) epigenetic imprinting via acetylation, methylation and emergence of non-coding (dys) regulatory RNAs ^26, 27^; (vii) extensive chemical damage to the extracellular matrix (ECM) by oxidation, nitrosylation, carbonylation, and cross-linking that alters its ability to maintain normal cell phenotype via appropriate adhesion kinase signaling ^28^; and (viii) the worsening of the above by breakdown of mucosal immunity, resulting in recurrent viral infections and bacterial colonization ^29^. Furthermore, mouse models have delineated important candidate pathways in the development of hypoplastic small lungs with reduced alveolarization that may predispose to early-onset disease. Key pathways implicated in these aberrations are the Wnt-Beta catenin system and the interplay of transforming growth factor-beta (TGFβ) isoforms with differential signal transduction via signal-transducing intermediates in the TGFβ system (SMADs) ^27^.

**Figure 2.**
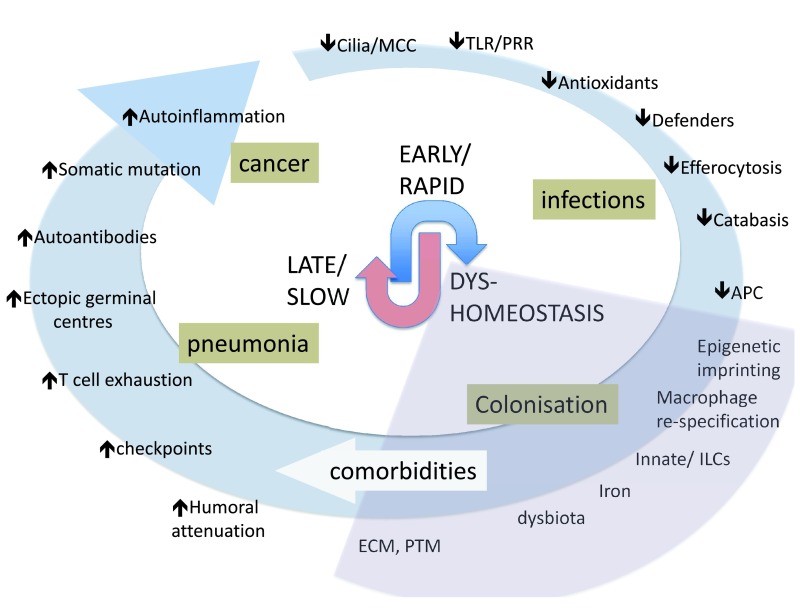
Smoke damages immunity from almost the first exposure. However, no single mechanism accounts for the deep and widespread impairment of immunity. Instead, the figure shows the interplay of EARLY/RAPID events, a period of DYS-HOMEOSTASIS where key damping feedback mechanisms and defenses are impaired, enabling LATE/SLOW processes that mark intractable inflammation and damage. These are notionally arranged, clockwise, as a temporal progression, but the exact sequence is not known. Ciliostasis and impaired mucociliary clearance (MCC) are prominent immediate effects of smoke, which also induces oxidant stress and inactivates/depletes anti-oxidants, allowing a large number of host defense pathways such as Toll-like receptor (TLR) and other pattern recognition receptors (PRRs) to be impaired. Similarly, a wide range of non-specific innate defense molecules, such as collectins and defensins (collectively called “defenders” here) become damaged. Macrophages quickly have their phagocytic capacity impaired for bacteria and dead cells (efferocytosis), contributing to impaired catabasis (resolution). Antigen-presenting cells (APCs) become hypofunctional. Chemical stressors epigenetically imprint tissue, and macrophages are redirected to an aberrant “M1lo-M2partial” maladapted phenotype. Innate cells and lymphocytes (ILCs) function poorly. Errors in iron handling promote further oxidation (e.g., via Fenton reactions) and promote bacterial growth as iron availability is rate-limiting. Colonization occurs in many patients. Extensive chemical modification of extracellular matrix (ECM) alters the tissue microenvironment, and internal post-translational modification (PTM) of cell proteins triggers stress and misfolded protein reactions attenuating key signaling pathways. At this point, comorbid conditions may become apparent. Later, B-cell humoral immunity wains, compounding normative immune aging; immune checkpoint blockers are increased, attenuating viral host defense; T cells become exhausted; and ectopic germinal centers form, promoting autoantibodies. The chemical stress on epithelium leads to somatic mutations, promoting auto-inflammation and, in a smaller group of patients, malignant transformation.

The second pathological feature is bronchiolitis, where the smaller airways become inflamed and fibrotic, again limiting airflow. There is important recent evidence from cross-sectional studies that very early in the course of COPD natural history in a dramatic destructive “pruning” of total small airways may occur well before overt changes in lung function measured by FEV1 occurs ^30^. Because total resistance to airflow depends on the total cross-sectional area of these small airways, this may contribute to permanent and intractable airflow limitation. Much less attention has been given to this area, mostly because mice do not have airway structures directly analogous to human small bronchioles. Nevertheless, the recent focus on epithelial-mesenchymal transition mechanisms based on advances in cancer research suggests that this process is active in COPD, driven by smoke, and involved in small airway fibrosis (see Figure 2) ^31^. Similarly, the interplay of gp130 and SMADs, signaling intermediates for IL-6 family cytokines and TGFβ, respectively, is implicated in both airway fibrosis and emphysema ^32, 33^. Both of these pathways also drive cancer progression and alteration in ECM composition. Although it has not been formally demonstrated for COPD, even subtle changes in ECM can drive forward cancer risk by altering cell phenotypic signaling via focal adhesion kinases and mechanosignaling components such as YAP/TAZ (a Yes-associated protein/transcriptional coactivator with PDZ-binding motif), transcription factors in the Hippo signaling system ^34^.

COPD is also often associated with bronchitis, the excessive production of mucus, which may be purulent (infected), especially during exacerbations. This is due to two processes: expansion of mucous glands which are found only in the larger cartilage-containing airways, and trans-differentiation of the smaller airway epithelium away from ciliated and non-secretory epithelial cell phenotypes toward a pattern rich in mucus-secreting goblet (mucus) cells. The molecular regulation of growth of mucous glands, greatly informed by breast cancer research on milk duct development, and the control mechanisms of epithelial progenitor/stem cell lineage regulation and epithelial transdifferentiation are areas of important progress ^35, 36^. Unsurprisingly, IL-13 and epidermal growth factor (EGF)-related signaling pathways are strongly implicated ^37^, but it is not yet known whether EGF receptor tyrosine kinase (EGF-RTK) or IL-13 (or IL-4Ra) inhibitors are effective. Recent genetic studies also point to genetic control of hypersecretion in COPD ^38^.

## The “comorbidome”

These classic descriptors of the lung pathology of COPD do not capture the full extent of the health burden because COPD is consistently associated with comorbidities. Given that the structural damage in COPD cannot be reversed with current technology and current treatments are purely symptomatic, there is great need to diagnose and treat chronic concomitant diseases that are modifiable by treatment (cardiovascular diseases, diabetes, osteoporosis, “myodebilitation” [musculo-skeletal impairment], cancer, and so on). About half of all patients with COPD die from a disorder of the heart and circulation, especially right heart failure, pulmonary hypertension, and thromboembolic disorders such as stroke, myocardial infarct, and deep vein thrombosis (DVT) (the incidence of which increases markedly after exacerbations). Moreover, COPD can be viewed as the lung expression of a “multi-morbidome” which extends to CNS affective disorder, metabolic derangement, renal disease, cancer (especially lung cancer), and musculoskeletal impairment ^39^. The role of systemic inflammation as a driver of the “comorbidome” is suggested by increases in systemic inflammatory biomarkers ^40^. The first US Food and Drug Administration (FDA)-approved biomarker for COPD interventional trials, fibrinogen, which tracks with increased cardiovascular risk ^41^ and predicts hospitalization and all-cause mortality in COPD, was approved in July 2015 after 6 years of work by the COPD Biomarker Qualification Consortium (CBQC). The broad hope is that future COPD drugs, perhaps systemic anti-inflammatory agents, that reduce fibrinogen correlate with better cardiovascular system (CVS), exacerbation, and mortality outcomes. Recent research demonstrating that defects in endothelial stem cells secondary to DNA damage (via oxidative stress?) and induced senescence, both implicated in emphysema progression, also occur in CVS disease in COPD provides a molecular basis for CVS comorbidity ^17^. The first major attempt in this direction, the SUMMIT trial (Study to Understand Mortality and Morbidity in COPD), which involved 16,485 patients and tested the hypothesis that combination therapy (fluticasone furoate, a glucocorticosteroid, and vilanterol, a LABA) might reduce all-cause mortality, was recently competed. Although this trial (available only as a press release at the time of this writing) failed to reach statistical significance on its primary and secondary endpoints, the investment in trials of this scale and ambition will shape the terrain and expectations of future COPD interventions ^42^.

## Impediments to clinical translation: the early disease problem

Despite this depth of basic knowledge, clinical translation in COPD has been extremely disappointing to date. At the macro level, there are several major impediments to progress. Firstly, despite excellent basic science, we do not understand COPD clinical endotypes. Endotypes are the underlying molecular pathways that drive clinically observed phenotypes ^43^. A given clinical phenotype (e.g., emphysema) may result from very distinct endotype pathways; as discussed above, multiple emphysema pathways have been found in preclinical model systems. Whereas we are now moving toward endotyping asthma into molecularly defined subgroups with matched precision medicines ^44^, COPD languishes in a phase of loose clinical phenotypic clustering, such as the new GOLD 4 quadrant ABCD system, that is pragmatic but imprecise. Secondly, as most COPD is diagnosed very late into its course, the currently approved FDA clinical trial outcomes are difficult to achieve; for example, current regulations require improvement in FEV1, a lung function parameter that is almost entirely insensitive to early changes in small airways. Recent clinical trials have therefore favored exacerbations as a primary outcome because, although loosely defined as “worsening of a patient’s condition requiring a change in usual care”, they are quantitative and can be linked to economic costs. In contrast there are no approved outcome measures for clinical trials that might instead try to target early disease. Critically, there is currently no FDA-approved way to study potential benefits of new drugs/therapies in early or “pre”-COPD ^45^. Two other CBQC “biomarkers”—the St George’s Respiratory Questionnaire (SGRQ) and the 6-minute walk test (6MWT)—have very recently achieved FDA-approval as trial endpoints. Other tools such as the widely used BODE (a multidimensional grading system that evaluates body mass index, measure of airflow obstruction, dyspnea score, and exercise capacity) index, the COPD Assessment Test (CAT), and EXACTPRO (a COPD exacerbation measurement tool) are not accepted as FDA biomarkers. These tests are invaluable but are weighted to dysfunction in advanced disease and are probably not sensitive to early disease. Lastly, COPD trials are almost the opposite of cancer, the field where precision medicine is most impressive in delivering new treatments. In cancer, drugs made for defined molecular lesions can be tested in pre-selected molecularly stratified patients and validated quickly against relatively easy and rapid trial endpoints, such as tumor size and progression-free survival. Accordingly, cancer trials are booming. COPD lacks endotypes, matched precision drugs, and rapid surrogates.

Thus, there is a profound need to develop trial strategies for early disease interventions that use molecular endotype stratification and have relatively quick surrogate outcomes that meet the rigorous standards demanded by regulatory authorities. What then are the most promising areas for translational medicine research in COPD?

## Lung function trajectories

In the late 1970s, Fletcher and Peto described an accelerated decline in lung function in current smokers and its flattened trajectory on cessation, cementing a dominant paradigm of COPD progression ^46^ which has subsequently been expanded ^47^. In 2015, Lange and colleagues questioned whether the normal decline in lung function that occurs with aging might lead to COPD if initial lung function levels were low ^48^. By combining three independent cohort studies (Framingham Offspring Cohort, Copenhagen City Heart Study, and Lovelace Smokers Cohort), they demonstrated unequivocally that low initial lung function (<80% FEV1 predicted assessed at age 40) leads to COPD in later life. In the same study, about half of those with normal lung function at age 40 had a rapid decline in lung function (53 ± 23 ml/year) and developed COPD, whereas the rest had slower decline despite similar smoke exposure. This study has several major implications. Firstly, at the public health level, it will be important to develop interventions to maximize lung function very early in life: known impediments to lung growth begin *in utero* and include infection, maternal smoking, poor maternal nutrition and post-partum exposure to irritants, poor nutrition, and low physical activity. Secondly, “rapid decliners” represent a prime target for early intervention in COPD prophylaxis studies. The accelerated decline may even start earlier because the study by Lange and colleagues used FEV1 as the lung function measure, which, though well suited to population studies, is quite insensitive to small airway changes. In independent studies, Hogg and colleagues have proposed that the primary lesion in COPD is the loss of small airways, which may precede development of emphysema of a grade sufficiently overt to impair FEV1 ^30^.

Two other studies point to other strategies for possible early intervention in COPD. Jensen and colleagues reported on disease “trajectories” in a 6.2 million-patient-strong database, assessing where the temporal relationship between codified health-care utilization could be assessed ^49^. Their data point to interesting patterns in which a COPD diagnosis is often preceded by a cluster of angina, atherosclerosis, and type II diabetes. More importantly, the post-diagnosis trajectories very frequently involve chest and other infections (cystitis and septicemia). A further important trajectory was muscle weakness, which can cause falls leading to life threatening fractures—especially when compounded by osteoporosis which is also very common in COPD. Using a similar strategy to question what precedes COPD, Jones and colleagues retrospectively examined a large UK general practice cohort and found a striking increase in both the number of courses of prescribed antibiotics for respiratory tract infections and chest x-rays (presumptive pneumonias), the frequency of which rose exponentially beginning around 18 years prior to COPD diagnosis suggesting that impairment of mucosal immunity occurs early in the natural history of COPD ^50^.

These data suggest two strategies: early life intervention to maximize lung structure and function and disease-modifying interventions in adults, ideally in the very early and “presymptomatic” phase of disease. Both will be extremely difficult because there are no agreed surrogate endpoints to enable clinical trials of reasonable duration. Furthermore, we lack safe and proven methods to detect early damage to alveoli and small airways. However, recent progress in high-resolution computed tomography (CT) imaging, which can detect benefits in alpha-1 anti-trypsin (A1AT) replacement therapy, provides a way forward, at least in adults.

## Impaired mucosal and systemic immunity

These studies also point to the much underestimated impairment of mucosal and systemic immunity that proceeds hand-in-hand with the development of COPD, underlying the increased propensity for viral and bacterial respiratory tract infections, colonization, and possibly cancer and autoimmune disease risk ^29^. At least 30% of patients with COPD are colonized, and recent metagenome studies suggest the percentage is probably higher ^51, 52^. Intuitively, colonization of the lung might reasonably be expected to drive chronic inflammation; however, this has been an area of controversy because isolates of *Haemophillus influenza* obtained from *stable* disease are not markedly pro-inflammatory in comparison with isolates obtained during a new exacerbation ^53^. This suggests adaption and even a near-commensal status for long-term colonizing pathogens. In one of the most elegant studies of recent years, Hogg and colleagues questioned whether the altered metagenome in COPD does indeed drive innate immunity and pro-inflammatory pathways. They extracted RNAs from archived lung and concurrently sequenced the metagenome and the host transcriptome, allowing the clear host response pattern to be matched with objective evidence of infection *in situ* ^54^.

There are two major implications for future therapy. Firstly, the simple strategy of trying to suppress the excessive inflammation in COPD with anti-inflammatory drugs or mediator-specific inhibitors of inflammation, an idea borrowed from the highly effective use of ICS, will be fraught with difficulty because, as has already been seen with anti-tumor necrosis factor biologicals ^55^ and the increased pneumonia risk that is a dose-proportional risk with ICS use ^56– 60^, the main consequence is increased infection risk rather than clinical improvement. This has already led to great interest in stratifying (restricting) ICS use in COPD to patients with elevated blood eosinophils, the same target group for treatment with eosinophil targeting anti-IL-5 or IL-5R-alpha antibodies that show glimmers of promise ^61^. Secondly, we must instead develop new drugs to restore mucosal immunity.

## Catabasis?

Therefore, a much better future strategy, it is argued here, is to find ways to restore defective immunity. One viable strategy is to recapitulate catabasis (resolution) by the use of specialized pro-resolving mediators such as resolvins and lipoxins which dampen inflammation but increase host defense ^62^ and are defective in COPD ^63^. However, at least for lipoxin A4, the vast amounts of serum amyloid protein A (SAA) released from pathogenic macrophages and other sources may allosterically impair ALX, the lipoxin A4 receptor ^64^. Another viable strategy is to target the very aberrant development and functional inactivation of lung macrophages. Schultze and colleagues ^65^ recently reported compelling integrative system transcriptomic data demonstrating that host defense functions in COPD macrophages are almost entirely inactivated, providing a molecular architecture to explain functional defects first observed by Hodge and colleagues ^66^. Given that the molecular regulation of pathogenic macrophage phenotypes is increasingly better understood, it may prove possible to redirect lineage commitment without impairing host defense or worsening inflammation ^20, 67– 71^. Lack of type 1 interferons (e.g., interferon beta [IFNβ]) predisposes COPD patients to viral infection, and inhalation repletion studies show some possibilities in experimental viral challenge models ^72^, but it is not known whether this pathway also promotes resolution.

## Targeting “myodebilitation”

Exacerbations of COPD leading to hospitalization are arguably the most important drivers of deterioration and overall economic cost and represent a major clinical trial outcome measure. As bed rest, steroid use, and inflammatory burden all compound to weaken already-frail patients, hospital stay itself is a risk factor for costly readmission. However, there are few predictors of which patients are more likely to need readmission which would be useful to structure targeted care, such as strength and conditioning training. Kon and colleagues have recently validated a new assessment tool, the 4-minute gait speed test (4MGS), as a simple predictor ^73^ of frailty and likelihood of readmission ^74^ but this index has not been qualified for regulatory purposes. Similarly, recent advances in high-resolution CT imaging of COPD have confirmed that markedly damaged lung architecture is a readmission risk ^75^. These articles point to an important area of emerging research, which is the interface of the COPD lung with “myodebilitation”, also reflected in osteopenia/poris and increased fall risk, which compound risk of life-threatening fractures ^76^.

Several interventions have demonstrated the possibility of at least partially reversing the loss of muscle strength and mass, which occurs more commonly than widely appreciated. One very attractive strategy is to target the activin type II receptor (bimagrumab, BYM338), which represses muscle growth when activated by myostatin, activin, and other growth/differentiation factors that are over-produced in COPD. This strategy has proven effective in increasing human skeletal muscle size and, if combined with physiotherapy, might mitigate “myodebilitation” ^73, 76, 77^. Other strategies include activating the ghrelin axis and insulin-like growth factor-1 (IGF-1) repletion, notwithstanding possible side-effect liabilities.

## Asthma chronic obstructive pulmonary disease overlap syndrome

**Figure 3.**
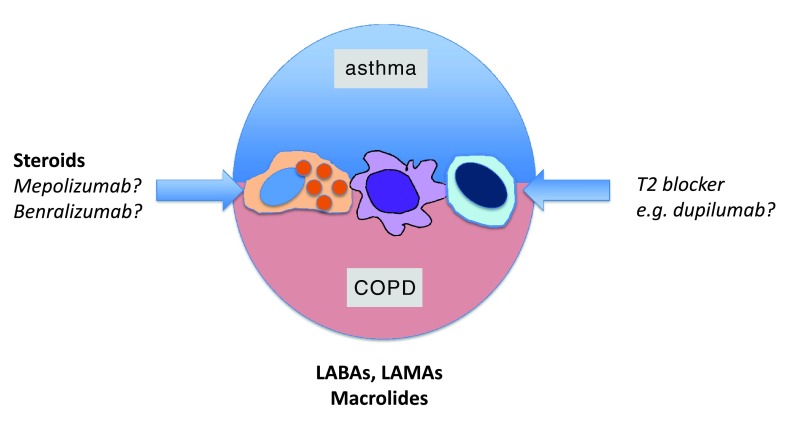
Asthma COPD overlap syndrome (ACOS) is a very poorly defined set of clinical presentations having some features of both asthma and COPD. There is good evidence that eosinophils are a clinically relevant trait as inhaled steroids are effective in patients with eosinophil-high ACOS, and anti-eosinophil monoclonal antibodies (e.g., mepolizumab, anti-IL-5, benralizumab, and anti-IL-5 receptor) have shown some promise to reduce exacerbations in COPD/ACOS in early clinical trials. ACOS has a clear T2-biased gene signature on profiling studies, but it is not yet known whether T2 blockers such as dupilumab (anti-IL-4 receptor alpha), which show promising efficacy in phase III clinical trials in asthma, will also be of benefit in ACOS. Interesting macrolides, which probably target the altered metagenome in both conditions, may also be effective in some patients with ACOS. Bronchodilators such as long-acting beta-2 adrenoceptor agonists (LABAs) and long-acting muscarinic antagonists (LAMAs) provide symptomatic relief but do not alter disease processes. Names in bold denote level A or B clinical trial evidence. Names in italics refer to early phase II clinical trial evidence or speculation based on mode of action. COPD, chronic obstructive pulmonary disease; IL, interleukin.

Lastly, it is worth considering the fraught category of ACOS. Early epidemiological studies in The Netherlands in the 1960s suggested that traits classically associated with asthma, specifically bronchial hyper-reactivity and atopy, were risk factors for progressive airflow limitation in COPD, sparking an enduring debate on differential diagnosis. Currently, there is intense debate on whether ACOS is a distinct entity, an expression of both asthma and COPD in the same patient given that up to 30% of asthmatics smoke, or an iatrogenic condition given that steroid treatment in severe asthma, which can have partially irreversible airflow limitation, kills eosinophils (the putative asthma mediator) but spares neutrophils (the putative COPD mediator). Patients with ACOS often have more severe disease, more airway involvement, and less emphysema. One intellectually interesting subtheme in the ACOS debate is to move away from the terms “asthma and COPD” ^35, 78, 79^ and instead concentrate on disease traits, also recently termed “treatable traits”, a concept well aligned with endotyping strategies ^43^. A second and pragmatic aspect is that ACOS provides a rationale to treat COPD patients with elevated eosinophils with ICS to minimize pneumonia risk and allocate expensive future biological medicines currently in development such as dupilumab (IL4Ra), mepolizumab (IL-5), and benralizumab (IL5Ra), see Figure 3. ^80, 81^. It has been suggested that ACOS is part of a disease spectrum ^82^, and clinical clustering studies using sputum profiling support overlapping ACOS, COPD, and asthma biomarker clusters ^83^, but the evidence for a molecular spectrum is not strong. Consistent with earlier pediatric epidemiology studies which found that severity level in asthma was set very early in life, longitudinal studies in adults point to ACOS being a fixed airflow consequence of more severe lifelong asthma ^84^. Asthma is classically not associated with emphysema and loss of elastic recoil, but Nadel and colleagues have recently reported a case series of non-smoking asthmatics with micro-centrilobular emphysema ^85^. An early genome-wide association study (GWAS) suggested several shared candidate asthma and COPD susceptibility loci, but these were not replicable ^86^. However, IL-13 has been implicated in both asthma and COPD ^87^, and direct profiling of genomic signatures in the mucosa of patients with COPD has unequivocally identified a T2 (TH2) gene signature in a subset of patients (that was not revealed by classic history-taking) showing elevated T2 biomarkers ^88^. An important caveat of these studies is that gene expression is quite fluid in COPD, especially in ICS-treated patients ^89^. Similarly, chitinases, which are classically induced by T2 signals, are elevated in more severe COPD. More recent GWAS’ of ACOS have identified single-nucleotide polymorphisms in candidate genes ^90^. It is also not known whether the T2-high gene signature observed in ACOS is mediated by classic TH2 CD4 ^+^ lymphocytes or, more likely, ILC2 innate lymphoid cells, an important matter because of their differential (re)activation and pharmacological profiles. Tissue damage, whether by smoke or viruses, releases substantial amounts of IL-33, an alarmin able to concurrently reactivate both ILC2 cells linked to T2 cytokines, and a separate macrophage and natural killer cell effector arm linked to IFNγ and IL-12, “Th1”-cytokines, as elegantly demonstrated by Humbles and colleagues ^91^. In the same study, however, active smoke exposure paralyzed ILC2 cells, favoring an exaggerated T1 response ^91^. Collectively, therefore, ACOS appears to have a molecular basis that, on present evidence, is best considered a co-expression of T2 immunity in the context of COPD.

This short review is by no means a comprehensive survey of COPD and its science over the last several years. It has instead lionized certain emerging themes that stress the need to transition COPD research away from clinical phenotyping toward molecular endotyping and, most critically, to develop the clinical research tools and systems needed to intervene much earlier in disease, when lung function is preserved and pathogenic processes are most likely to be tractable to therapy. In late disease, the importance of frailty and “myodebilitation”, as well as impaired immunity, have been emphasized as tractable and highly attractive fields of future research.

## Abbreviations

ACOS, asthma chronic obstructive pulmonary disease overlap syndrome; CBQC, COPD Biomarker Qualification Consortium; COPD, chronic obstructive pulmonary disease; CNS, central nervous system; CT, computed tomography; CVS, cardiovascular system; ECM, extracellular matrix; EGF, epidermal growth factor; FDA, US Food and Drug Administration; FEV1, forced expiratory volume in 1 second; GWAS, genome-wide association study; ICS, inhaled glucocorticosteroid; IFN, interferon; IL, interleukin; ILC2, type 2 innate lymphocyte; LABA, long-acting beta-2 adrenoceptor agonist; LAMA, long-acting muscarinic antagonist; SMAD, signal-transducing intermediates in the transforming growth factor beta system; TGFβ, transforming growth factor beta.
